# Towards definitive management of allergic rhinitis: best use of new and established therapies

**DOI:** 10.1186/s13223-020-00436-y

**Published:** 2020-05-27

**Authors:** Lubnaa Hossenbaccus, Sophia Linton, Sarah Garvey, Anne K. Ellis

**Affiliations:** 1 Department of Biomedical and Molecular Sciences, Queen’s University, Kingston, Canada; 2 Department of Medicine, Queen’s University, Kingston, Canada; 3 Allergy Research Unit, Kingston General Health Research Institute, Kingston, Canada

**Keywords:** Allergic rhinitis, Pharmacotherapy, Antihistamines, Nasal steroids, Leukotriene receptor antagonists, Allergen-specific immunotherapy, Combination therapy, Treatment algorithm

## Abstract

**Background:**

Allergic rhinitis (AR) is an inflammatory disease of the nasal mucosa impacting up to 25% of Canadians. The standard of care for AR includes a treatment plan that takes into account patient preferences, the severity of the disease, and most essentially involves a shared decision-making process between patient and provider.

**Body:**

Since their introduction in the 1940s, antihistamines (AHs) have been the most utilized class of medications for the treatment of AR. First-generation AHs are associated with adverse central nervous system (CNS) and anticholinergic side effects. On the market in the 1980s, newer generation AHs have improved safety and efficacy. Compared to antihistamines, intranasal corticosteroids (INCS) have significantly greater efficacy but longer onset of action. Intranasal AH and INCS combinations offer a single medication option that offers broader disease coverage and faster symptom control. However, cost and twice-per-day dosing remain a major limitation. Allergen immunotherapy (AIT) is the only disease-modifying option and can be provided through subcutaneous (SCIT) or sublingual (SLIT) routes. While SCIT has been the definitive management option for many years, SLIT tablets (SLIT-T) have also been proven to be safe and efficacious.

**Conclusion:**

There is a range of available treatment options for AR that reflect the varying disease length and severity. For mild to moderate AR, newer generation AHs should be the first-line treatment, while INCS are mainstay treatments for moderate to severe AR. In patients who do not respond to INCS, a combination of intranasal AH/INCS (AZE/FP) should be considered, assuming that cost is not a limiting factor. While SCIT remains the option with the most available allergens that can be targeted, it has the potential for severe systemic adverse effects and requires weekly visits for administration during the first 4 to 6 months. SLIT-T is a newer approach that provides the ease of being self-administered and presents a reduced risk for systemic reactions. In any case, standard care for AR includes a treatment plan that takes into account disease severity and patient preferences.

## Background

### Allergic rhinitis

Allergic rhinitis (AR) is an IgE-mediated inflammatory disease of the nasal mucosa, triggered by exposure to airborne allergens. It is estimated to afflict almost 25% of Canadians [1] and has a significant impact on sleep, work, and school performance. AR is often associated with atopic dermatitis, food allergy, and asthma; this allergic disease progression known as the atopic march [2]. Symptoms primarily include rhinorrhea, nasal blockage, and sneezing, though ocular symptoms can also occur. In Canada, AR tends to be classified as either seasonal (SAR) or perennial (PAR) [3].

Standard of care for AR includes a treatment plan that considers patient preferences, the severity of the disease, and most essentially involves a shared decision-making process between patient and provider. Diagnosing AR and finding a care plan should consist of in-depth patient history, physical exam, and skin test to confirm allergies [4]. The patient’s history should include evaluating nasal and ocular symptoms such as rhinorrhea, nasal itching, sneezing, allergic conjunctivitis, and nasal congestion [3, 4]. The timing of the onset of symptoms is essential in determining which allergens are suspect. A comprehensive review of concomitant medications such as nonsteroidal anti-inflammatory drugs, angiotensin converting enzyme (ACE) inhibitors, beta-blockers, and intranasal decongestants helps diagnose or rule out other causes of rhinitis [5]. Concomitant atopic diseases such as asthma must be assessed as up to 40% of patients with allergic rhinitis, also have asthma [6]. A family history of atopic disease is a strong predictor that supports a diagnosis of AR and is important to include in the patient’s history [7]. A physical exam, including evaluating the nasal mucosa for swelling and/or nasal polyps and an oropharynx exam for signs of postnasal drip, are both useful. A simple observation of the patient is valuable in finding signs of AR, such as observing for allergic shiners, mouth breathing, throat clearing, and transverse nasal crease. Skin and chest exams are important diagnostic tools to look for other atopic diseases such as eczema and asthma. Although a thorough history and physical exam are useful, there is no one specific finding that is pathognomonic for allergic rhinitis [4]. Therefore, skin prick testing (SPT) for suspect aeroallergens should be performed. SPT is quick, inexpensive, and a minimally invasive way to confirm or rule out allergies [4, 5]. In vitro specific IgE testing may be used where SPT is not available or when it is not feasible due to eczema, dermatographism, or if the patient is unable to stop medications with antihistaminic activity. Before deciding on pharmacotherapy or immunotherapy, all patients must be provided information on how to reduce or eliminate exposure to their allergens [5].

The standard treatment algorithm for AR begins with allergen avoidance [4]. Patients are encouraged to limit exposure to relevant allergens by taking precautionary measures, such as closing windows to prevent pollen entry, maintaining humidity < 40% in homes to prevent dust mite and mold growth, and/or using high-efficiency particular air (HEPA) filters to remove animal dander from the air. If symptoms persist despite avoidance strategies, newer generation oral antihistamines (AHs) are the first-line pharmacologic option. They are the most commonly used treatment method for AR, being safe, and efficacious. Intranasal corticosteroids (INCS) are also recommended as first-line treatments, and in fact, show greater efficacy than AHs. Combination intranasal therapies featuring antihistamines and corticosteroids also exist, such as azelastine hydrochloride/fluticasone propionate (AZE/FP) and can provide more significant relief. Patients who remain symptomatic despite INCS or combination therapies, or those who do not wish to stay on such treatments on a long term basis, or pediatric patients in whom primary prevention of the development of asthma symptoms is a priority [8, 9], should be offered allergen-specific immunotherapy (AIT). AIT is the only disease-modifying option and can be provided through subcutaneous (SCIT) or sublingual (SLIT) routes. While SCIT has been the definitive management option for many years, SLIT tablets (SLIT-T) have also been proven to be safe and efficacious. This review aims to present the facts and recommended guidelines for the treatment algorithm of AR.

### Oral antihistamines

For decades, AHs have been the most utilized class of medications for the treatment of AR. AHs are inverse agonists; that is, they target H1 receptors (H1 antihistamines) at binding sites that are different from those of histamine [10]. There are two generations of oral antihistamines (first-, and newer-generation AHs), with newer-generation AHs being an improvement of their predecessor. First-generation AHs, such as diphenhydramine are associated with adverse central nervous system (CNS) side effects, including sedation and mental impairment, as well as anticholinergic side effects such as dry mouth, dry eyes, urinary retention, and constipation [11]. Newer generation H1-antihistamines are safer than first-generation agents and should be the first-line antihistamines for the treatment of allergic rhinitis [12]. However, for reasons that are discussed elsewhere, both patients and practitioners continue to select first-generation AHs [13]. This section aims to review the recognized risks of first-generation AH and to explore recent advances in newer generation AHs.

#### Adverse effects of first-generation AHs

The adverse effects associated with first-generation AHs have been reported since their introduction in the 1940s [14]. Currently, it is well-known that these drugs have poor receptor selectivity and can bind non-selectively to several receptors in the body, including antimuscarinic-, anti-serotonin-, and anti-α-adrenergic receptors as well as cardiac potassium channels [15].

First-generation AHs can also cross the blood–brain barrier (BBB) and bind H1-receptors on neurons throughout the CNS and, therefore, may cause drowsiness, sedation, somnolence, and fatigue leading to impairment of cognitive function, memory, and psychomotor performances [16]. The strong sedative qualities of older, first-generation AHs are why they are used as sleep aids. Paradoxically, the same dose is utilized to promote sleep as is used to relieve rhinitis symptoms [13].

Long-term, randomized, controlled studies of the safety of first-generation antihistamines are limited. However, many studies outline the association of these drugs with transportation-related injuries and fatalities. A recent review of toxicology tested profiles from 6677 fatally injured civil aviation pilots in the US from 1990 to 2012. In this study diphenhydramine was the most common drug found on autopsy capable of causing impairment (7.3%) [17]. As a result, first-generation AHs are now banned for use by commercial and military pilots before or during flights [12].

The CNS effects of first-generation AHs resemble and exacerbate those produced by alcohol and by other CNS-active chemicals. It may not be surprising, then, that diphenhydramine and other first-generation antihistamines are common drugs of abuse [18]. Infants and children who experience accidental or intentional overdose may present with paradoxical excitation, including irritability, delirium, respiratory depression, and coma [16, 18, 19].

Cardiac toxicity was previously an under-recognized risk of first-generation AHs. Diphenhydramine and hydroxyzine interfere with cardiac potassium channels involved in action potential repolarization. As a consequence, these drugs may cause dose-related prolongation and a form of polymorphic ventricular dysrhythmia called ‘torsade de pointes’ [20–22].

#### Safety of newer generation AHs

Newer generation antihistamines came on the Canadian market in the 1980s. These newer non-sedating AHs were developed to be less soluble, limiting their ability to penetrate the BBB [15]. Since their introduction, many randomized controlled clinical trials, including off-label trials, have evaluated their safety. Prescription-event monitoring studies in England comparing the risk of drowsiness and sedation between newer-generation antihistamines involving thousands of allergic individuals have proven there is a low risk of sedation for cetirizine, desloratadine, fexofenadine, levocetirizine, and loratadine [23, 24]. Even accidental exposures of up to 30-fold ingestions of cetirizine, loratadine, and fexofenadine did not result in any serious adverse events [25, 26].

It is worth mentioning that two second-generation AHs, astemizole and terfenadine, have been associated with prolonged cardiac AT intervals and “torsade de pointes” at high doses. This was a specific issue with these compounds and are not representative of a class effect of second-generation agents. Both drugs have been off the market for over 20 years [12].

#### Efficacy of first-generation AHs vs. newer generation

It is a misconception that older AHs have a faster onset of action than newer agents. In a double-blind placebo-controlled trial comparing cetirizine and loratadine to chlorpheniramine (a first-generation AH), both cetirizine and loratadine were found to have a significantly faster onset and a longer duration of action [27].

Many well-powered randomized, double-blind, placebo-controlled clinical trials have compared the efficacy of newer generation antihistamines [28–33]. Studies using the Environmental Exposure Unit (EEU) have shown that cetirizine and terfenadine have superior efficacy over loratadine and astemizole [33]. Likewise, cetirizine significantly reduces symptoms compared to both loratadine and placebo, with an onset of action of 60 min [29]. Cetirizine and fexofenadine, both OTC antihistamines, have been further examined in the EEU. In these studies, cetirizine had a longer duration of action than fexofenadine [32, 34].

New advances in antihistamines available on the Canadian market have focused on rupatadine and bilastine. Rupatadine is a novel substance which, in addition to being an H1 antagonist, is also a potent platelet-activating factor (PAF) inhibitor [35]. A randomized, placebo-controlled, double-blind study was conducted at four medical institutions in Japan [36]. Nine hundred patients were randomly assigned to placebo, rupatadine 10 mg, or rupatadine 20 mg. The rates of adverse effects were 6.6%, 14.1%, and 15.0% for placebo, rupatadine 10 mg, or rupatadine 20 mg, respectively. Somnolence was most frequently reported in rupatadine 20 mg (7.3%) and rupatadine 10 mg (7.0%) [36]. There have been several studies comparing the 10- and 20-mg doses of rupatadine with the approved daily doses of ebastine, levocetirizine, and cetirizine showing rupatadine to be beneficial [37–39].

Bilastine is another newer generation antihistamine that is highly selective for the H1 receptor, has a fast onset of action and a long duration of action. In a double-blind, randomized, placebo-controlled, balance four-treatment, four-period crossover phase II study using the Vienna Challenge Chamber, the efficacy of bilastine (20 mg), cetirizine (10 mg) and fexofenadine (120 mg) to relieve SAR symptoms were compared [40]. All treatments were significantly more effective (p < 0.001) than placebo in reducing total nasal symptom score (TNSS), without significant difference between the three antihistamines during the first 4 h after administration. Bilastine at 20 mg was as effective as cetirizine 10 mg and fexofenadine 120 mg in terms of onset of action and in reducing eye symptoms 1 h after the intake. Bilastine was still effective 26 h after the intake, confirming the prolonged duration of action [40].

In summary, first generation antihistamines are no longer recommended for the treatment of allergic rhinitis [13]. Newer agents such as cetirizine, loratadine, desloratadine, fexofenadine, rupatadine and bilastine have all demonstrated superior efficacy over placebo with an enhanced side effect profile, and should be chosen as first-line agents for AR. A summary of these newer agents for clinical use can be seen in Table 1.

**Table 1:** Detailed summary of approved pharmacotherapy options available in Canada

Product name	OTC/ prescription	Indication	Dosage	Onset of action	Duration of action	Toxicity	Pregnancy category
Histamine H1 receptor antagonist							
Bilastine (^Pr^BLEXTEN™)	Prescription	Treatment of nasal and non-nasal symptoms of SAR in patients Treatment of symptoms of CSU in patients	1 × 20 mg tablet qd	1 h	26 h	Abdominal pain, dizziness, and headache	Insufficient data at this time
Cetirizine hydrochloride (REACTINE^®^)	OTC: 10 mg Prescription: 20 mg*	Treatment of nasal and non-nasal symptoms of SAR, PAR Treatment of symptoms of CSU	1−2 × 5 mg tablets qd 1x 10 mg tablet qd 1× 20 mg tablet qd 1× 10 mg capsule qd 5–10 mL of syrup 5 mg/5 mL qd (2–12 yo) 10 mL of syrup 5 mg/5 mL qd (> 12 yo)	20−60 min	Up to 24 h	Somnolence, headache, and dry mouth	B
Desloratadine AERIUS^®^	OTC	Treatment of nasal and non-nasal symptoms of SAR, PAR Treatment of symptoms of CSU	1× 5 mg tablet qd (> 12 yo) 2.5–5 mL of syrup 0.5 mg/mL qd (2–12 yo)	75 min	24 h	Dry mouth and headache	C
Fexofenadine ALLEGRA^®^	OTC	Treatment of symptoms of SAR, PAR and CSU	1× 60 mg tablet q12h (12 h formulation) 1 × 120 mg tablet qd (24 h formulation)	2–3 h	12 h	Dizziness, drowsiness, and dry mouth	C
Loratadine CLARITIN^®^	OTC	Treatment of symptoms of SAR and PAR Treatment of symptoms and signs of CSU and other dermatologic disorders	1–2 × 5 mg tablets qd (> 12 yo) 1 × 10 mg tablet qd (> 12 yo) 5–10 mL of oral solution 1 mg/mL qd (2–12 yo)	2 h	Up to 24 h	Fatigue, headache, dry mouth, sedation, gastrointestinal disorders such as nausea, gastritis, and also allergic symptoms like rash	B
Loratadine CLARITIN^®^	OTC	Treatment of symptoms of SAR and PAR Treatment of symptoms and signs of CSU and other dermatologic disorders	1−2× 5 mg tablets qd (> 12 yo) 1 × 10 mg tablet qd (> 12 yo) 5–10 mL of oral solution 1 mg/mL qd (2–12 yo)	2 h	Up to 24 h	Fatigue, headache, dry mouth, sedation, gastrointestinal disorders such as nausea, gastritis, and also allergic symptoms like rash	B
Histamine H1 receptor antagonist platelet activating factor receptor antagonist							
Rupatadine (^Pr^RUPALL™)	Prescription	Treatment of symptoms of SAR, PAR and CSU	1 × 10 mg tablet qd (≥ 12 yo) 2.5 mL of oral Solution, 1 mg/mL qd (2–11 yo with body weight of 10-25 kg) 5 mL of oral solution, 1 mg/mL qd (2-11 yo with body weight of > 25 kg)	1–2 h	Up to 24 h	Somnolence, headache, tiredness, asthenia, dry mouth, nausea, and dizziness	Insufficient data at this time
Intranasal antihistamines							
Levocabastine hydrochloride ^Pr^LIVOSTIN^®^	Prescription	Treatment of symptoms of SAR and PAR	2 sprays (0.5 mg/mL) EN 2qd (≥ 12 yo)**	10 min	24 h	Nasal irritation, epistaxis, somnolence, headaches, dizziness, eye irritation, dry mouth and tiredness	
Intranasal corticosteroids							
Beclomethasone dipropionate BECONASE^®^	Prescription	Treatment of SAR and PAR	2 sprays (50 mcg/metered dose) EN bid	1–2 weeks; max benefit 3–4 weeks	Unknown	Headache, nosebleed or blood-tinged mucus, burning or irritation inside the nose, sneezing or sore throat	C
Fluticasone propionate FLONASE^®^	OTC	Treatment of SAR and PAR	2 sprays (50 mcg/metered dose) EN qd or q12h for severe rhinitis (≥ 12 yo) 1–2 sprays (50 mcg/metered dose) EN qd (4–11 yo)	1–2 weeks; max benefit 3–4 weeks	Unknown	Headache, nosebleed or blood-tinged mucus, burning or irritation inside the nose, sneezing or sore throat	C
Mometasone furoate monohydrate ^Pr^NASONEX^®^	Prescription	Treatment of the symptoms of SAR and PAR Adjunctive treatment to antibiotics in acute episodes of rhinosinusitis in patients ≥12 years, where signs or symptoms of bacterial infection are present Treatment of symptoms of mild to moderate uncomplicated acute rhinosinusitis in patients ≥12 years, where signs or symptoms of bacterial infection are not present Treatment of nasal polyps in patients ≥18 year	2 sprays (50 mcg/metered dose) EN qd (≥ 12 yo) 1 spray (50 mcg/metered dose) EN qd (3–12 yo)	1–2 weeks; max benefit 3–4 weeks	Unknown	Headache, nosebleed or blood-tinged mucus, burning or irritation inside the nose, sneezing or sore throat.	C
Budesonide RHINOCORT^®^	Prescription	Treatment of SAR and PAR perennial, and vasomotor rhinitis unresponsive to conventional therapy Treatment of nasal polyps and in the prevention of nasal polyps after polypectomy	1–2 sprays (64 mcg/metered spray) EN q12h (≥ 12 yo)	1–2 weeks; max benefit 3-4 weeks	Unknown	Cough, throat irritation, hoarseness, bad taste, headache, nausea and dryness of the throat	B
Leukotriene receptor antagonist							
Montelukast SINGULAIR^®^	Prescription	Treatment and prevention of asthma Treatment of symptoms of SAR	1 × 10 mg tablet qd (≥ 15 yo) 1 4–5 mg chewable tablets qd*** (≤ 14yo) 4 mg oral granules qd (2–5 yo)	2 h	24 h	Abdominal pain and headache In rare circumstances, psychiatric side effects (insomnia, nightmares, suicidal ideation)	B
Antihistamine and Corticosteroid Agent							
Futicasone-azelastine DYMISTA^®^	Prescription	Treatment of moderate to severe SAR and associated ocular symptoms	1 spray (137 mcg/50 mcg per metered spray) EN q12hr (≥ 12 yo)	15 min	26 h	Dysgeusia, epistaxis, and headache	Insufficient data at this time

Health Canada. Drug Product Database. https://health-products.canada.ca/dpd-bdpp/index-eng.jsp. Accessed Dec 11, 2019

CSU, Chronic Spontaneous Urticaria; PAR, Perennial Allergic Rhinitis; SAR, Seasonal Allergic Rhinitis

* 20 mg tablet is prescription only

** It is not useful to continue the treatment for more than 3 days if no improvement is seen. There are no clinical trials to support continuous treatment duration of greater than 10 weeks

*** The dosage for pediatric patients 6 to 14 years of age is one 5 mg chewable tablet daily to be taken in the evening. The dosage for pediatric patients 2 to 5 years of age is one 4 mg chewable tablet daily to be taken in the evening or one packet of 4 mg granules to be taken orally once a day in the evening

### Intranasal antihistamines

One concern regarding oral antihistamines (OAHs) is the possibility that OAHs cannot reach high enough concentrations in the nasal mucosa following oral administration to inhibit histamine-stimulated cytokine release and other mediators of early- and late-phase allergic reactions. [41] Intranasal antihistamines (INAHs) ensure drug delivery to the nasal mucosa, enhancing local anti-allergic and anti-inflammatory effects while minimizing systemic exposure to therapy [42]. The 2016 ARIA guidelines recommend using intranasal antihistamines (e.g., olopatadine, and levocabastine) in intermittent but not persistent AR [3]. While azelastine (AZE) is the most well-studied INAH, it is not available in Canada. However, levocabastine hydrochloride nasal spray (LEVO), another INAH, is available in Canada (see Table 1 for clinical usage information) and has shown to be equivalent to AZE in terms of efficacy and safety. In a recent multicenter, randomized, double-blind, parallel-group trial, 244 patients with moderate-to-severe allergic rhinitis were randomized to receive either AZE (0.1%) or LEVO for 14 consecutive days. Statistically significant changes from baseline in TNSS were seen in both treatment groups. No significant differences were seen between the two groups in terms of evaluation of therapeutic effect, total effective rate, and onset of action, except for a higher symptom relief rate in the LEVO group than the AZE group within 30 min of administering the first dose. Adverse reactions were mild to moderate, with an incidence of 0.9% for LEVO and 2.5% for AZE [43].

In short, while intranasal antihistamines are safe and effective, only one is available in Canada and is often hard to obtain currently.

### Intranasal corticosteroids

ARIA guidelines recommend INCS as the best option for both mild and moderate to severe AR in both children and adults [3]. INCS inhibit the early and late-phase allergic in AR by preventing the recruitment of immune cells, and the release of inflammatory mediators from cells involved in the pathophysiology of AR [44–46]. Many INCS have been approved since the introduction of beclomethasone in the late 1970s [47]. All of the INCS currently available are efficient in controlling symptoms of AR, such as nasal congestion and itching, rhinorrhea, and sneezing [48]. To differentiate products involves factors such as cost, ease of dosing, and sensory issues, such as aroma and taste, which can affect patient preference [49]. As will be described in more detail below, the significant disadvantages of INCS are patient adherence and the length of time they take to reach maximal effect [50].

#### Safety of intranasal corticosteroids

INCS are less likely to display the systemic effects of oral steroids such as growth suppression, and ocular effects, due to reduced exposure and lower bioavailability. However, INCS are associated with mild to moderate local adverse effects. These include, epistaxis, nasal drying, burning, and stinging sensations [51].

The ability of INCS to suppress bone growth is controversial. Measurement of the hypothalamic–pituitary–adrenal (HPA) axis function is a sensitive way to evaluate the potential systemic effects of INCS. Using this method, a 1-year study showed that the use of beclomethasone dipropionate aqueous nasal spray twice daily resulted in significant suppression of growth in children compared with placebo [52]. Similar studies have shown no suppression of bone growth in children after 1 year of treatment with the recommended pediatric dose of mometasone furoate aqueous spray [52] or with budesonide [53]. It is important to recognize that additive exogenous steroid effects on the HPA axis can occur when INCS treatment accompanies concurrent INCS or other topical corticosteroids [54].

The literature examining the risk of development of glaucoma and/or cataracts from the use of INCS is also complex and controversial. While it is clear that inhaled and oral corticosteroid use is associated with high long-term risks of cataract development [55], the potential risk of cataracts with the use of nasal corticosteroids is more complex. Recently, a systematic review assessed whether the use of INCS is associated with increased intraocular pressure (IOP) above 20 mm Hg, glaucoma, or formation of posterior subcapsular cataracts in adult patients with rhinitis [56]. A total of 484 studies were identified with 10 randomized controlled trials meeting the inclusion criteria. Meta-analysis of 2226 patients revealed that the use of INCS is not associated with a significant risk of elevating IOP or developing a posterior subcapsular cataract in patients with allergic rhinitis. The absolute increased incidence of elevated IOP in patients using INCS compared to placebo was 0.8% (95% CI 0 to 1.6%). There were zero cases of glaucoma in both placebo and INCS groups at 12 months [56]. Future studies should formally evaluate for glaucoma rather than use IOP measures as a surrogate.

#### Efficacy of intranasal corticosteroids

Compared to placebo and antihistamines, INCS have significantly greater efficacy [57]. This is further demonstrated in a systematic review comparing the efficacy of INCSs and OAHs that analyzed 5 controlled trials with a total of 990 patients. INCS were superior to OAHs in improving total nasal symptoms score and in relieving nasal obstruction, rhinorrhea, nasal itching, sneezing, and quality of life mean difference. However, there was no difference in relief of ocular symptoms [58]. Similarly, Carr et al., compared the efficacy of AZE and fluticasone propionate (FP) in SAR via a post hoc analysis of data from a previously published direct-comparison study. FP was superior to AZE in alleviating rhinorrhea but AZE showed comparable efficacy for all other nasal and ocular symptoms. However, more patients treated with AZE achieved a 50% reduction from baseline in their ocular symptoms by day 14 compared with patients in the FP group and achieved this response up to 3 days earlier than FP [59].

To summarize, INCS are mainstay treatments for moderate to severe allergic rhinitis. All Health Canada approved products are generally safe and effective, and should be used with consideration to formulation, delivery device preferences and out of pocket costs to the patient (summarized in Table 1).

### Leukotriene receptor antagonists

The other major therapeutic class of drug indicated for AR therapy are the leukotriene-receptor antagonists (LTRAs). LTRAs block the activity of cysteinyl leukotrienes (CysLTs), a potent inflammatory mediator associated with nasal congestion, mucus production, and inflammatory cell recruitment responsible for AR symptoms [60]. Currently, the only LTRA available in Canada is montelukast (see Table 1). The current ARIA guidelines recommend an LTRA or an OAH for use in patients with SAR. It is also mentioned that the choice of and LTRA or OAH will mostly depend on patient preferences and local availability and cost of specific medications (conditional recommendation, moderate certainty of evidence). In patients with PAR, the guidelines suggest an OAH rather than a LTRA (conditional recommendation, low certainty of evidence) [3]. When compared with placebo, montelukast improves the disease-specific quality of life of patients with persistent AR [61]. In a 32-week randomized, placebo-controlled crossover study in patients with persistent AR, antihistamine treatment alone or in combination with montelukast was compared. Montelukast, alone or in combination with an antihistamine, gave a gradual increase in nasal symptom improvement within 6 weeks of treatment [62]. Similar results have been shown in patients with seasonal AR [63]. More recent studies have suggested the presence of neuropsychiatric side effects with the use of montelukast, and as such, the U.S. Food and Drug Administration has discouraged its use as a first-line therapy for mild AR [64].

### Intranasal antihistamine and intranasal corticosteroid combination

It is evident that no single medication class is without limitations (Table 1). The 2016 update of the ARIA guidelines does suggest (with low to moderate certainty) that combination treatment with an OAH or INAH and an INCS may be appropriate for patients with SAR [3]. Indeed—the concurrent use of an INCS and INAH has provided benefits over monotherapy in patients with moderate-severe SAR [65]. However, there are disadvantages to this approach, including a negative impact on concordance [66], increased runoff both posteriorly and anteriorly [67], and nonhomogeneous distribution of active agents on the nasal mucosa [68]. Thus, there is an obvious need for a single medication option which offers broader disease coverage, and faster symptom control.

Combining an INAH and an INCS, AZE/FP is a novel formulation in a single spray. There are many benefits to AZE/FP. Patients benefit from the additive effects that result from the different primary mechanisms of action of each drug (AZE and FP) and there is possible improvement in adherence to therapy by delivering the two agents in a single device [67]. Moreover, the single spray application provides more uniform distribution and greater retention in the nasal cavity than sequential sprays of AZE and FP [68]. Perhaps the most significant disadvantage to AZE/FP is that it requires twice per day dosing.

The efficacy and safety of AZE/FP have been assessed in several controlled clinical studies. One 14-day SAR study compared AZE/FP with formulation- and device-matched AZE and FP [69]. The AZE/FP combination provided greater overall nasal symptom relief than either FP, AZE, or placebo. More AZE/FP-treated patients achieved a 50% reduction in their overall nasal symptom burden. They did so many days earlier than those treated with FP or AZE. The combination had an onset of action of 30 min, and the clinical benefit was observed during the first day of assessment and sustained over the entire course of treatment [69]. AZE/FP was also compared to commercially available FP (Flonase generic) and AZE (Astelin^®^), respectively. The treatment difference was more considerable. When nasal and ocular symptoms were combined, AZE/FP was more than twice as effective as either FP or AZE. Likewise, patients reached a 50% reduction in their overall nasal symptom burden one week faster than those treated with FP or AZE [70]. The long-term safety of AZE/FP has been evaluated in subjects with PAR or vasomotor rhinitis. There were no safety findings that would preclude the long-term use of AZE/FP in the treatment of allergic rhinitis [71].

In patients who do not respond to INCS, a combination INAH/INCS should be considered, assuming cost is not prohibitive to the patient.

### Allergen specific immunotherapy

AIT is a treatment that provides the potential for long-term relief from AR [72]. It includes subcutaneous and sublingual methods of administration. As a potentially disease-modifying therapy, it is surprisingly often the last treatment option for patients whose symptoms are ill-managed by the traditional pharmacologic therapies, despite showing evidence for primary use. Indeed, it is an option for patients who have not responded to standard pharmacotherapy or those who wish to avoid the use of pharmacotherapy on a long-term basis. Factors such as adherence and comorbid conditions should be considered with young patients, as well as in the elderly. While there is evidence to support the efficacy of AIT in both populations, an individual assessment of the applicable risks and benefits should be taken into consideration. Contraindications for AIT in treatment for AR include patients with severe and uncontrolled asthma, comorbid heart conditions (such as high blood pressure), which require that use of beta-blockers, and caution should be used in the setting of concomitant ACE inhibitor therapies. The initiation of AIT during pregnancy is contraindicated due to the theoretical increased risk of anaphylaxis, though the continuation of therapy appears to be safe [73].

Protective effects from AR symptoms can be sustained for up to 2 years after 3 years of AIT, regardless of modality [74]. While a patient may be determined to be polysensitized to allergens through a skin prick test, AIT is suggested only for the allergens which manifest clinical symptoms. However, the presence of polysensitization does not limit the clinical benefit of the AIT being given to the seasonally or perennially relevant allergen [75–79].

### Subcutaneous immunotherapy

SCIT, known colloquially as “allergy shots”, is the classic method of providing AIT, featuring the injection of allergen underneath the skin of the upper arm. These injections are composed of diluted allergen extracts combined with phenol and glycerin preservatives [80]. SCIT involves a “build-up” phase, where increasing doses of allergen are given on a regular (usually weekly) basis until a determined effective dose is reached, which has been shown in clinical trials to be associated with the development of immunological tolerance. The maintenance phase follows, during which the patient continues to receive regular monthly injections. The Canadian Society of Allergy and Immunology has published an Immunotherapy Manual with the suggested effective doses to include in the maintenance dose [81]. The conventional treatment typically ranges from 3 to 5 years until patients note long-term symptom reduction or elimination, at which point, treatment is often stopped.

The efficacy of SCIT has been well-established for many allergens, including house dust mite [82], birch pollen [83], Timothy grass [84], and rye grass [85]. Adverse effects can occur with this treatment. Local adverse reactions are common in 26–86% of patients [85], and may include redness, irritation, or swelling at the site of injection, and can be managed through the use of oral antihistamines, topical corticosteroids, and ice packs applied at the site immediately following injection. The occurrence of systemic adverse reactions in patients undergoing SCIT ranges from 1 to 4% [81], observed typically within 30 min post-injection. The presentation of systemic reactions can vary from mild to severe, including anaphylaxis, classified as a Grade 1–5 system [86]. In an 8-year North American surveillance study, two fatalities as a result of adverse reactions were reported [87]. Due to the risk of systemic reactions associated with SCIT and the specialized route of administration, it must be administered in a physician’s office with rescue equipment readily available. A monitoring period of at least 30 min is required to ensure no complications as a result of treatment, and/or treat any complications that do ensue.

SCIT can be administered as the complete 3–5 year protocol or pre-seasonally. Pre-seasonal SCIT is a shorter course of treatment taken a few weeks before the start of the pollen season, offering short-term disease protection. The injections typically feature an aluminum hydroxide or microcrystalline tyrosine adjuvant to enhance the antigen-specific immune response [88]. If not followed up with traditional AIT treatment, the benefit of pre-seasonal SCIT is not long-lasting and would need to be re-administered annually. In a randomized, double-blind, placebo-controlled study, Mosges et al. investigated the safety and efficacy of a short course grass allergen SCIT pre-seasonal schedule. Over 3 weeks, 554 participants received 8 injections of either placebo or increasing grass allergen extract. The participants recorded a combined symptom and medication score throughout the pollen season and completed the standardized Rhinoconjunctivitis Quality of Life Questionnaire [89] before and during the pollen season. It was found that 92% of the participants completed treatment, and from this, it can be seen that short-course treatment before the onset of the pollen season was effective at reducing the symptoms of the patients [90].

While SCIT has proven to be beneficial in AR treatment, it is only received by 2–9% of affected patients in the US [91]. Practical challenges include weekly visits to the doctor’s office, a minimum 3-year treatment period, significant local reactions, and the potential for severe or possibly fatal systemic adverse reactions lead many patients to discontinue this treatment option. While SCIT remains the best option for definitive relief to date, novel techniques have been developed to further the options available for the treatment of AR.

### Sublingual immunotherapy

SLIT is a newer immunotherapeutic approach, requiring no injections, and instead involves the dissolution of allergen extract under the tongue, taken once daily for an extended period. The allergen extract is standardized and compressed into a tablet and can even be in aqueous form as drops; however, the latter approach has not been standardized nor approved by governing bodies, such as the FDA and Health Canada. In Canada, the current SLIT tablet (SLIT-T) options include Oralair^®^ (5 grass pollens), Grastek^®^ (timothy grass pollen), Ragwitek^®^ (short ragweed pollen), and Acarizax^®^ (house dust mite bodies and feces) (Table 2) [92]. The advantages of this treatment include convenience, as it can be administered at home, except for the first dose, which requires physician supervision, and a significantly reduced risk of severe systemic effects. Due to the self-administrative nature of SLIT-T, physicians need to provide patients with clear directives before starting treatment. Important instructions include: avoid eating and drinking for at least 5 min before and after administration, the treatment should be stopped for dental work or if open sores are present in the mouth, do not double up on doses that have been missed, and return to the clinic if 14 consecutive doses are missed.

**Table 2:** Detailed summary of approved sublingual immunotherapy options available in Canada

Product Name	Composition	Indications	Contraindications	Pregnant/Nursing Women	Dosage	Administration	Adverse Drug Reactions
Oralair^®^	Cocksfoot (*Dactylis glomerata*) Sweet vernal grass *(Anthoxanthum odoratum)* Rye grass *(Lolium perenne)* Meadow grass (*Poa pratensis*) Timothy grass (*Phleum pratense*)	Moderate to severe seasonal grass pollen AR suffered for at least two pollen seasons Between 5–50 years old Positive skin prick test and positive specific IgE titre to *Poaceae* grass pollen Unresponsive to conventional pharmacotherapies	Extreme sensitivity to the allergen based on prior anaphylactic experience under exposure β-blockers ACE inhibitors Severe/unstable asthma (FEV1 < 70%) Severe immune deficiency or autoimmune disease Malignant diseases (cancers) Oral inflammation	Should be used only if the potential benefit justifies the potential risk to the fetus and mother	100 IR* 300 IR*	Three-day escalation phase (Day 1: 1 × 100 IR; Day 2: 2 × 100 IR) followed by maintenance phase consisting of 1 × 300 IR until the end of treatment Treatment should be initiated 4 months before the onset of the pollen season and maintained throughout the season For adult patients (18–50 years old): discontinue if no improvement is seen after three seasons	Itching and swelling localized to the mouth and throat
Grastek^®^	Timothy grass (*Phleum pratense*)	Moderate to severe seasonal Timothy and related grass pollen induced AR Between 5–65 years old Clinically relevant symptoms for at least two pollen seasons Positive skin prick test and/or positive specific IgE titre to *Phleum pratense* Unresponsive to conventional pharmacotherapy	Hypersensitive to any non-medicinal ingredients Previously had severe systemic reaction to Timothy or related grass immunotherapy Unstable/severe chronic asthma (FEV1 < 70% after pharmacologic treatment; < 80% in children) β-blockers Active inflammatory conditions in the oral cavity	Treatment should not be initiated in pregnant women No clinical data are available for use during lactation	2800 BAU**	Treatment can be initiated at any time during the year but should be at least 8 weeks before the grass pollen season and maintain throughout the season First dose should be administered under the supervision of an experienced physician with a 30-min observation period One 2800 BAU tablet daily	Throat irritation
Ragwitek^®^	Short Ragweed (*Ambrosia artemisiifolia*)	18 to 65 years old Clinically relevant symptoms for at least two pollen seasons Positive skin prick test and/or positive specific IgE titre to *Ambrosia artemisiifolia* Unresponsive to conventional pharmacotherapy	Unstable/severe chronic asthma (FEV1 < 70% after pharmacologic treatment) Previous reaction to ragweed allergy shots, tablets, or drops β-blockers Active inflammatory conditions in the oral cavity Allergic to the non-medicinal ingredients	Treatment should not be initiated in pregnant women No clinical data are available for use during lactation	12 Amb a 1-U	Treatment should be initiated at least 8 weeks before the grass pollen season and maintain throughout the season First dose should be administered under the supervision of an experienced physician with a 30-min observation period One 12 Amb a 1-U tablet daily	Throat irritation Itching of the mouth, ears, and eyes Swelling or numbness of the mouth
Acarizax^®^	House Dust Mites (*D. farinae and D. pteronyssinus*)	18 to 65 years old Moderate to severe house dust mite-induced allergic rhinitis Positive skin prick test and/or positive specific IgE titre to *D. farinae* or *D. pteronyssinus* Unresponsive to conventional pharmacotherapy	Severe/unstable asthma Previous reaction to house dust mite allergy shot, tablets, or drops Beta-blockers Swelling or sores in mouth Mouth injury or surgery If diagnosed with eosinophilic esophagitis Allergic to the non-medicinal ingredients	Treatment should not be initiated in pregnant women No clinical data are available for use during lactation	12 SQ-HDM	Treatment can be initiated at any time during the year First dose should only be taken in the doctor’s office, followed by a 30-min monitoring period One 12 SQ-HDM tablet daily	Throat irritation Itching, burning, or tingling of the mouth Swelling of the lips or tongue

* Index of reactivity

** Bioequivalent allergy units

The use of SLIT has been characterized for use in grass pollens [93], ragweed [79], and other allergens [94]. The efficacy of SLIT is similar to that of SCIT. In a systematic review by Elliott et al., it was found that in comparison to placebo, SCIT and SLIT were both more effective than placebo, and resulted in similar quality of life scores [95]. In a recent evaluation of AIT in patients afflicted with allergic conjunctivitis, significant improvements (p < 0.05) were seen clinically, though no significant difference was observed between the SCIT and SLIT modes of administration [96]. The use of dual allergen SLIT tablets (grass and ragweed) are well tolerated [97]. In an investigation by Ortiz et al., the use of single allergen and multiallergen SLIT was investigated in polysensitized patients. While symptom scores decreased with treatment, no significant differences were observed between the number of allergens included in the treatment regimen [98].

**Fig. 1:**
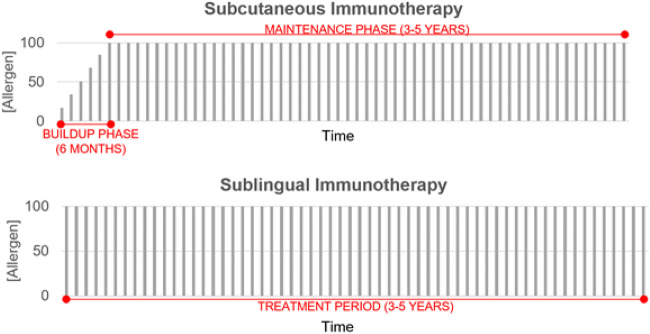
Relative comparison of the dosing schedule for subcutaneous (SCIT), sublingual (SLIT), and intralymphatic (ILIT) immunotherapies Adapted from Senti et al. (2019) [105]

In comparison to SCIT, SLIT has a less worrisome safety profile, as systemic reactions are rare, and no fatalities have been reported. Adverse local reactions are common for the first 2 weeks of treatment, often localized to the oral cavity, and have been seen to subside within 30 to 60 min [99]. Both SCIT and SLIT are disease-modifying, with effects persisting for years after treatment [74, 100]. Treatment for less than 2 years has been found not to provide protective effects, whereas, at 1 year of treatment, SCIT appears to be more beneficial than SLIT. Importantly, however, after 2 years of treatment, the symptomatic effects of both methods are equal [101]. Thus, AITs require a minimum time commitment of 3 years (Fig. 1), an important consideration for patients considering this treatment option. In an investigation of the costs associated with a 3-year house dust mite AIT treatment in Canada, while SCIT had a lower upfront cost, the total savings were more considerable with SLIT [102]. Similarly, Ellis et al. investigated whether Timothy grass SLIT treatment would confer protection against birch pollen AR. In assessing symptom scores, no significance was established, suggesting that SLIT is allergen specific [103].

### The future of AIT

Within the past decade, there have been different immunotherapy techniques that have come under investigation, such as intralymphatic immunotherapies (ILIT). ILIT proposes the injection of allergens directly into lymph nodes guided by ultrasound. In the literature, this technique thus far appears to be safe, effective, and requires a lesser time commitment, however, it has not yet been standardized or approved for clinical use. [104].

## Conclusion

There is a range of available treatment options for AR that reflect the varying disease length and severity. The standard treatment algorithm begins with allergen avoidance, followed by pharmacologic agents. For mild to moderate AR, newer generation AHs should be first-line treatments and preferred over older AHs, due to better safety profiles. INCS are mainstay treatments for moderate to severe AR, with the Health Canada approved products proven to be safe and effective. Therefore, the formulation, delivery device preferences, and out of pocket costs to patients must be weighed. In patients who do not respond to INCS, a combination of intranasal AH/INCS (AZE/FP) should be considered, assuming that cost is not a limiting factor. The only disease-modifying option for patients who do not respond to or wish to avoid long-term use of pharmacotherapy is AIT. SCIT and SLIT-T both require a minimum three-year treatment period to provide long-term symptom relief; however, the mode of delivery and possible adverse effects do differ. While SCIT remains the option with the most available allergens that can be targeted, it has the potential for severe systemic adverse effects and requires weekly visits for administration during the first 4 to 6 months. SLIT-T is a newer approach that provides the ease of being self-administered and presents a reduced risk for systemic reactions. In any case, standard care for AR includes a treatment plan that takes into account patient preferences, disease severity, and is a shared decision-making process between patient and provider.

## Data Availability

Not applicable.

## Abbreviations

ACE: Angiotensin converting enzyme
AH: Antihistamine
AIT: Allergen-specific immunotherapy
AR: Allergic rhinitis
AZE: Azelastine
AZE/FP: Azelastine hydrochloride/fluticasone propionate
BBB: Blood brain barrier
CNS: Central nervous system
CysLT: Cysteinyl leukotriene
EEU: Environmental exposure unit
FP: Fluticasone propionate
HPA: Hypothalamic pituitary adrenal axis
ILIT: Intralymphatic immunotherapy
INAH: Intranasal antihistamine
INCS: Intranasal corticosteroid
IOP: Intraocular pressure
LEVO: Levocabastine hydrochloride
LTRA: Leukotriene receptor antagonist
OAH: Oral antihistamines
PAF: Platelet-activating factor
PAR: Perennial allergic rhinitis
SAR: Seasonal allergic rhinitis
SCIT: Subcutaneous immunotherapy
SLIT: Sublingual immunotherapy
SLIT-T: SLIT-tablet
SPT: Skin prick testing
TNSS: Total nasal symptom score
